# Mechanism and Application of Developmental Factors in Plant Genetic Transformation

**DOI:** 10.3390/ijms262010135

**Published:** 2025-10-18

**Authors:** Lixin Zhang, Fang Wang, Biao Luo, Na Chen, Yan Wang, Xianwen Zhang

**Affiliations:** Institute of Virology and Biotechnology, Zhejiang Academy of Agricultural Sciences, Hangzhou 310021, China; zhanglixin_1994@163.com (L.Z.); ffang0125@163.com (F.W.); biaoluo@stu.hunau.edu.cn (B.L.); 15857179687@163.com (N.C.)

**Keywords:** callus formation, developmental factors, genetic transformation, shoot regeneration, transformation efficiency

## Abstract

Genetic transformation serves as a critical tool for gene function research and crop improvement. However, its efficiency is often low and highly dependent on species, genotypes, and explant types, significantly restricting its broader application. Many developmental factors have been proven pivotal not only for plant growth and development but also for the regulation of callus formation and shoot regeneration, which are key steps in the process of genetic transformation. Thus, this review focuses on the application of developmental factors in enhancing transformation efficiency across species. Developmental factors are classified into four regulatory pathways: morphogenesis, wound signaling, epigenetic modification, and hormone signaling. Among them, morphogenic factors have been extensively studied for enhancing transformation efficiency, while the potential of the other three pathways remains less explored in species beyond *Arabidopsis*. We summarize these established mechanisms and regulatory networks, providing a valuable reference for elucidating these mechanisms in other plant species. Furthermore, we also propose strategies for identifying species-specific efficient developmental factors and improving molecular mechanisms. Ultimately, this review provides a comprehensive summary of the mechanism and application of developmental factors and offers theoretical support to overcome the bottlenecks in genetic transformation.

## 1. Introduction

Climate change, increasing human population, and the scarcity of land and water resources threaten global food security, posing unprecedented challenges to modern crop improvement [1,2]. Although traditional breeding approaches have significantly increased agricultural productivity over the past century, they now face fundamental limitations in addressing contemporary multidimensional challenges [3,4,5]. In this critical context, Jiayang Li proposed the intelligent breeding of smart crops as the core of Breeding 5.0 generation [6]. Smart crops refer to improved crop varieties that dynamically adapt to environmental fluctuations, improving yield, quality, and stress resilience while reducing resource inputs. Intelligent breeding integrates advanced biotechnology (e.g., genome editing and multi-omics) with information technology (e.g., AI and big data) to systematically decode agronomic traits and enable precision crop design in elite varieties. As the key technology of Breeding 5.0, genetic transformation serves as the essential delivery platform for both gene editing tools (e.g., CRISPR-Cas9) and overexpression constructs, enabling precise genomic modification and controlled gene expression in plants. Genetic transformation not only enables functional gene studies to decipher agronomic trait mechanisms but also allows the targeted genetic improvement of elite germplasms, thereby accelerating the development of precision-designed smart crops [7]. Therefore, enhancing genetic transformation efficiency is crucial for the realization of these potentials.

The plant transformation process generally involves two sequential steps: the induction of callus formation from explants on auxin-rich callus-inducing medium (CIM) followed by shoot regeneration after transfer to cytokinin-enriched shoot-inducing medium (SIM) [8]. In many crop species, callus formation is typically initiated at wound sites of explants [9]. The efficiency of callus formation and shoot regeneration is highly dependent on hormones, species, genotype, and explant types [8,10,11,12]. Model plants such as *Arabidopsis* and tomato (*Solanum lycopersicum*) consistently achieve high transformation efficiency with various explant types, including leaves, roots, and floral tissues [13,14,15,16,17]. In contrast, essential cereals such as maize (*Zea mays*) and wheat (*Triticum aestivum*) show significantly lower transformation efficiency. These species rely on immature embryos as explants, a process that is not only labor-intensive and time-consuming but also requires strictly controlled conditions [10,17,18,19]. Substantial evidence indicates that many elite varieties are particularly recalcitrant to genetic transformation. This is exemplified in wheat, whose transformation efficiency ranges from just 2.7% in the commercial variety Jimai22 to 45.3% in the model genotype Fielder. Notably, agriculturally important varieties such as Aikang58 and Jing411 have failed to produce transgenic plants [20,21]. Similarly, in soybean (*Glycine max*), model genotypes like Jack, Williams82, Dongnong50, and Tianlong1 are commonly used for genetic transformation, while elite commercial varieties such as Heihe43 and Zhonghuang13 have rarely been transformed successfully [22,23,24,25,26,27].

Numerous studies have demonstrated that developmental factors have emerged as powerful targets for overcoming the limitations of plant genetic transformation [28,29,30,31,32]. This review systematically examines the application of these developmental factors in enhancing transformation efficiency across diverse plant species. Meanwhile, we also summarize well-characterized regulatory pathways of callus formation and shoot regeneration in *Arabidopsis*. Additionally, we discuss strategies to decode the regulatory networks controlling genetic transformation in other plants and identify efficient developmental factors. These insights will facilitate understanding the underlying mechanism and help overcome existing bottlenecks in plant genetic transformation.

## 2. Morphogenic Factors

### 2.1. WOX

WUSCHEL-related homeobox (WOX) homeodomain transcription factors, including WUSCHEL (WUS) and WOX, act as regulators of plant growth and development by controlling stem cell fate in meristems [33,34,35,36,37]. In particular, *WOX5* is a key regulator of the pluripotency acquisition of callus [38,39].

In wheat, the application of *TaWOX5* dramatically improved transformation efficiency with less genotype dependency [21]. Overexpression of *TaWOX5* achieved transformation efficiency of up to 94.5% in the readily transformable wheat varieties CB037 and Fielder. More importantly, it also greatly improved the transformation efficiency in the recalcitrant variety Jimai22 from 5.8% to 55.4%. This variety is China’s most widely cultivated wheat variety, with an annual planting area exceeding 2 million hectares. Although immature embryos from stressed wheat plants typically failed to produce viable embryonic calli, the expression of *TaWOX5* remarkably overcame this barrier, achieving 33.8% transformation efficiency in the CB037 [11,21]. Furthermore, *TaWOX5* overexpression induced distinctive phenotypic markers (wide/short flag leaves and thickened stems) in transgenic wheat plants, enabling visual screening of both marker-free transgenic and transgene-free edited plants.

While the potential of *WOX* genes to boost transformation efficiency in other species is underexplored, their function in regulating callus initiation and shoot regeneration has been experimentally validated. In maize, overexpression of *ZmWOX2a* promoted transformation in the recalcitrant genotype B73 [40,41]. WOX transcription factors regulate organ regeneration by modulating auxin biosynthesis and cytokinin (CK) responsiveness, which are both critical for establishing cellular pluripotency [39]. For example, in apple (*Malus domestica*), MdWOX11 was found to bind the promoter of *MdCKX5* to induce its expression, leading to CK degradation and the subsequent downregulation of CK-responsive genes that collectively suppress adventitious shoot formation [42]. These findings suggest that a systematic exploration of *WOX* family members in other crops could enable breakthrough gains in transformation efficiency with less genotype dependency in crop improvement.

### 2.2. BBM-WUS

*BABY BOOM* (*BBM*), an AP2/ERF domain transcription factor, functions as a key activator of cell proliferation and morphogenesis during somatic embryogenesis [43]. WUS, a homeodomain protein, is a master regulator of embryogenic and meristematic stem cells [33,44,45,46,47].

Research on the *BBM/WUS*-mediated enhancement of transformation efficiency has primarily been conducted in maize. *ZmBBM* expression significantly increases callus transformation efficiency, whereas *ZmWUS2* expression stimulates somatic embryo formation but inhibits shoot regeneration [48]. The *ZmBBM-WUS2* combination further enhanced transformation efficiency beyond that achieved by either gene alone, but it induced developmental abnormalities and sterility in transgenic plants. To obviate these adverse pleiotropic effects in T0 plants, several strategies have been developed. One approach employed the maize phospholipid transferase protein promoter (*Zm*-*PLTP_pro_*) to drive *ZmBBM* expression, along with the *ZmWUS2* expression cassette under control of the nopaline synthase promoter (*Nos_pro_*) or a maize auxin-inducible promoter (*Zm-Axig1_pro_*) [49]. This system enabled tissue- and timing-specific expression, thereby alleviating pleiotropic effects of *ZmBBM-WUS* co-expression. Alternatively, developmentally regulated promoters, such as *Ole*, *Glb1*, *End2*, and *Ltp2*, have been used to drive an inducible site-specific recombinase (*Cre*) to excise morphogenic genes after transformation but before regeneration [50]. A novel alternative, known as the “altruistic” transformation system, employed *ZmWUS2*-stimulated somatic embryogenesis in neighboring cells [51]. This method utilized two *Agrobacterium* strains: one carrying a *ZmWUS2* expression cassette on a binary vector and the other carrying vectors harboring selectable and visual markers. When the two *Agrobacterium* strains were mixed at a ratio of 9:1 (selectable marker: *ZmWUS2*) and used to infect immature embryos, transient *ZmWUS2* expression stimulated somatic embryogenesis in neighboring cells containing the selectable marker. This method not only improved the transformation efficiency but also increased the frequency of the CRISPR/Cas-targeted genome editing in sorghum (*Sorghum bicolor* L.) [52]. An optimized version of this “altruistic” system was developed by introducing a lethal gene element into the binary vector carrying *ZmWUS2* [53]. The optimized system achieved an average transformation efficiency of 19.5%, representing a 2.5-fold increase over the conventional methods. Notably, the typically recalcitrant variety Zheng58 exhibited a high transformation efficiency of 24.0%. In addition, all these methods enhance transformation efficiency while obviating the adverse pleiotropic effects in transgenic plants; the “altruistic” and the lethal-gene-based systems also offer operational simplicity.

Moreover, the expression of *ZmBBM* and *ZmWUS2* has been shown to enhance the transformation efficiency of immature embryos in other monocots, including rice (*Oryza sativa*), sorghum, sugarcane (*Saccharum officinarum*), and wheat [48,54]. Importantly, *BBM-WUS* enabled the direct *Agrobacterium*-mediated transformation of mature seed-derived embryo axes or leaf segments, bypassing the need for a callus or meristem culture step in maize and sorghum [48,55]. Consequently, this advance eliminates the dependence on immature embryo quality for maize genetic transformation, enabling year-round experimentation. The application of *BBM-WUS* not only significantly enhances transformation efficiency but also substantially simplifies the operational workflow.

### 2.3. GRF-GIF

GROWTH-REGULATING FACTORs (GRFs) are sequence-specific DNA-binding transcription factors that form functional complexes with GRF-INTERACTING FACTORs (GIFs) to regulate plant growth and developmental processes [56,57]. *GRFs* are involved in the regulation of the transition between stem cells to transit-amplifying cells and callus proliferation during organ development, while GIFs boost the transcriptional activity of GRFs [58,59,60]. Due to this central role in regulating cell proliferation, GRFs or GRF-GIFs have been demonstrated to increase transformation efficiency in both monocots and dicots.

In monocots, the wheat *TaGRF4-GIF1* chimera boosted the transformation efficiency up to 65.1% in the tetraploid variety Kronos, 63.0% in the tetraploid wheat Desert King, and 61.8% in the hexaploid variety Fielder, which was significantly higher than that of *TaGRF4* and *TaGIF1* alone [61]. The chimera also enhanced callus formation in rice and induced regeneration independent of exogenous CK, enabling the generation of selectable marker-free transgenic plants [61]. Similarly, *TaGRF4-GIF1* and *DcGRF4-GIF1* chimeras enhanced *D. catenatum* regeneration from meristematic tissues of young seedlings, bypassing callus induction and antibiotic selection [62]. In dicots, the watermelon (*Citrullus lanatus*) *ClGRF4-ClGIF1* chimera boosted regeneration efficiency to 47.0% in the wild variety and achieved >19.6% efficiency in previously non-transformable genotypes compared to 5.2% in the control and even enabled the production of diploid seedless varieties [63]. The soybean *GmGRF3-GIF1* chimera significantly enhanced transformation across diverse genotypes, with transformation efficiencies of 5.5% in Suinong14 and 13.8% in Suinong4, 2.7-fold higher than for the empty vector control [23]. *GRF-GIF* chimeras from *citrus* and grape (*Vitis vinifera*) enhanced citrus epicotyl regeneration, achieving a 4.7-fold increase in efficiency compared to empty vector controls [61]. While grape *GRF4-GIF1* and *AtGRF5* enhanced the regeneration of leaf-petiole explants in cassava (*Manihot esculenta*), they did not stimulate the regeneration of friable embryogenic callus [64]. *AtGRF5* has also been shown to enhance transformation in sugar beet (*Beta vulgaris* ssp. *vulgaris*), improve transgenic shoot regeneration in soybean and sunflower (*Helianthus annuus*), and promote transgenic callus cell proliferation in canola (*Brassica napus*) [65]. Moreover, *GRF-GIF* chimeras from tomato, pepper (*Capsicum annuum*), *citrus*, and grape consistently enhanced shoot regeneration efficiency across diverse lettuce genotypes [66].

The *GRF-GIF* chimera enhances transformation efficiency across species, with *miR396*-resistant fusions exhibiting the strongest effects. *miR396* post-transcriptionally represses *GRF*, but modifying its target sites in *GRF* transcripts enhances transformation efficiency and plant growth in many species by stabilizing *GRF* expression [23,61,62,63,66,67]. Co-cultivation with dual strains (r*GRF-GIF* (microRNA-resistant *GRF-GIF*) + the gene of interest) and selection only for the gene of interest significantly enhanced shoot regeneration efficiency, resulting in regenerated plants without r*GRF-GIF* [66]. Unlike *BBM-WUS*, which induces developmental defects, the *miR396-GRF/GIF* system enables the efficient regeneration of fertile, phenotypically normal transgenic plants without requiring specialized promoters or transgene excision. Thus, the *miR396*-*GRF*/*GIF* signaling module has recently emerged as an alternative to *BBM-WUS* chimeras for enhancing transformation across a range of crop species.

### 2.4. DOF

DNA binding with one finger (DOF) proteins are a group of plant-specific transcription factors involved in diverse developmental processes and environmental responses [68,69,70]. DOF transcription factors modulate auxin and CK signaling response and are in turn transcriptionally induced by abscisic acid (ABA), auxin, and CK [68,71].

Studies show that wounding induces the expression of four DOF transcription factors (*HIGH CAMBIAL ACTIVITY2* (*HCA2*)/*DOF5.6*, *TARGET OF MONOPTEROS6* (*TMO6*)/*DOF5.3*, *DOF2.1*, and *DOF6*), thereby promoting tissue regeneration [72]. In wheat, overexpression of *TaDOF5.6* and *TaDOF3.4* enhanced transformation efficiency from 26.0% to 50.0% and 55.0% in Fielder, while achieving 29.0% and 22.0% in Jimai22, and 34.0% and 17.0% in Kenong199, respectively [73]. In addition, transgenic plants overexpressing these genes grow normally without developmental defects, suggesting their potential utility in the genetic transformation across a range of crop species. In *Arabidopsis*, *DOF3.4* promoted periclinal divisions in the cortex and epidermis, while *DOF5.6/HCA2* enhanced radial growth in the procambium [74,75]. Given that these processes are fundamentally linked to cell proliferation, we propose that *TaDOF3.4* and *TaDOF5.6* improve regeneration in wheat by promoting cell proliferation.

Beyond *TaDOF5.6* and *TaDOF3.4*, there is no evidence that other *DOFs* directly increase genetic transformation efficiency. Other *DOF* genes are known to regulate cell cycle progression, which suggests their potential involvement in callus formation and shoot regeneration. Further research is needed to elucidate the role of these *DOFs* in regulating transformation efficiency. For instance, OBF Binding Protein 1 (OBP1) shortens G2-S transition of the cell cycle by binding to the promoter of *CYCD3.3* and *AtDOF2.3*, leading to dwarf plants in *Arabidopsis* [76]. OBP1, DOF1.1/OBP2, and OBP3 stimulate OBF4/DOF5.4 binding to the *ocs* element, which is also induced by auxin, salicylic acid (SA), and CK [71]. *OBP4/DOF5.4* is a negative regulator of G2-M phase transition of the cell cycle and cell expansion, resulting in the dwarfish phenotype [77]. Similarly, *OBP4/DOF5.4* inhibits cell elongation and promotes pericycle cell division during callus formation [78]. Additionally, *VASCULAR-RELATED DOF1* (*VDOF1*)*/Dof4.6* and *VDOF2/Dof1.8* play roles in vascular cell differentiation in *Arabidopsis* [79]. Interestingly, PEAR proteins, including PHLOEM EARLY DOF 1 (PEAR1)/DOF2.4, PHLOEM EARLY DOF 2 (PEAR2)/DOF5.1, DOF1.1/OBP2, DOF3.2/DOF6, DOF5.6/HCA2, and DOF5.3/TMO6, regulate radial growth in the procambium and move across short ranges via plasmodesmata [75]. These findings show that DOF proteins can move between cells and provide a new direction for functional studies of other regulatory proteins.

### 2.5. Combination of Morphogenic Factors

*BBM-WUS* promotes de novo somatic embryogenesis, whereas *GRF-GIF* chimeras primarily facilitate the regeneration of later-stage mature somatic embryos. The integration of these two chimeras therefore produces synergistic effects, substantially enhancing regeneration efficiency in recalcitrant genotypes. In the study on wheat, co-transformation was employed to mix *Agrobacterium* strains harboring either a vector with individual constructs containing *BBM-WUS*, *GRF-GIF*, a combination of *BBM-WUS* and *GRF-GIF*, or *WOX5* [54]. Individual expression of *WOX5*, *BBM-WUS*, or *GRF-GIF* increased transformation efficiency from 10.6% to 28.7%, 29.3%, and 38.7%, respectively. Notably, the combined expression of *BBM-WUS* and *GRF-GIF* demonstrated synergistic effects, significantly increasing transformation efficiency to 55.4%. In maize, *GRF-GIF-BBM* increased the transformation efficiency to 26.0–37.0%, a significantly higher efficiency than that of controls [80,81,82]. The combination did not show detectable adverse effects, indicating its utility in practical research.

## 3. Wound-Inducible Factors

In standard tissue culture protocols, explants (isolated plant tissues) rather than intact plants are commonly employed as the initial material. Wounding of explants induces diverse cellular responses, including the synthesis of plant hormones, the interruption of cell-to-cell communication, and the disruption of long-distance signaling, ultimately leading to callus formation at wound sites. Callus formation directly determines shoot regeneration and even transformation efficiency.

### 3.1. WIND1

In *Arabidopsis*, the AP2/ERF-type transcriptional regulator *WOUND-INDUCED DEDIFFERENTIATION1* (*WIND1*) and its homologs *WIND2*, *WIND3*, and *WIND4* are induced upon wounding and play a key role in promoting callus formation [83,84]. *AtWIND1* increases cellular plasticity in explants of *Brassica napus* and *Arabidopsis*, which subsequently promotes shoot regeneration [84]. Similarly, in maize, *ZmWIND1* significantly enhanced shoot regeneration and improved transformation efficiency to 37.5% in Xiang249 and 16.6% in Zheng58 [85]. WIND1 directly binds to the promoter of *ENHANCER OF SHOOT REGENERATION1* (*ESR1*) to activate its expression. *ESR1*, another AP2/ERF transcription factor, promotes both callus formation and shoot regeneration in response to wounding by upregulating the expression of *CUP-SHAPED COTYLEDON1* (*CUC1*), a shoot regeneration regulator [86]. Additionally, *WIND1* upregulated B-type *RESPONSE REGULATOR* (*ARR*)-mediated CK response, further promoting cell dedifferentiation [83].

### 3.2. REF

Wounding induces plant elicitor peptide REGENERATION FACTOR1 (REF1), a well-characterized signaling ligand. REF1 binds to its receptor PEPR1/2 ORTHOLOG RECEPTOR-LIKE KINASE 1 (PORK1) to activate the expression of *SlWIND1* [87]. SlWIND1 also binds to the promoter of the REF1 precursor gene, establishing a positive feedback loop that amplifies regenerative responses. Additionally, REF1 can boost regeneration and transformation efficiency across multiple recalcitrant species. SIREF1 enhanced the transformation efficiency of recalcitrant varieties *S. peruvianum* PI126944 and *S. habrochaites* LA1777 by 6-fold and 12-fold. In soybean, GmREF1 significantly enhanced transformation efficiency by 2-fold in conventional gene transformation and by 5-fold in genome editing, respectively. Similarly, application of TaREF1 increased the regeneration and transformation efficiency of Jimai22 by 8- and 4-fold, respectively. ZmREF1 enhanced the regeneration and transformation efficiency of B104 by 6- and 4-fold. Collectively, these results underscore the broad potential of REF1 to improve the transformation efficiency in both dicot and monocot species. Moreover, biotinylated REF1 peptide can be commercially synthesized and directly supplemented into the culture medium. This strategy greatly facilitates the application of genome editing and conventional gene transformation technologies in plant research and crop improvement.

### 3.3. PLT

In addition to *WIND1*, the AP2/ERF transcription factors *PLETHORA3* (*PLT3*), *PLT5*, and *PLT7* are activated by wounding and contribute to callus formation at wound sites [88]. For instance, *Agrobacterium tumefaciens* injection tests showed that *AtPLT5* promoted callus formation and transformation in snapdragon (*Antirrhinum majus*), *Brassica rapa*, and tomato, although it was ineffective in sweet pepper (*Capsicum annuum*) [89]. Specifically, *AtPLT5* achieved a transformation efficiency of 11.3% in snapdragon and 13.3% in tomato. In *Arabidopsis*, *PLT3*, *PLT5*, and *PLT7* promote shoot regeneration by sequentially regulating *PLT1* and *PLT2* at the early stage and subsequently controlling *CUC1* and *CUC2* expression at the later stage [90]. Furthermore, PLT1 and PLT2 interact with WOX5 and WOX7 to activate *L-Tryptophan aminotransferase of Arabidopsis1* (*TAA1*), leading to increased auxin biosynthesis [39,91].

### 3.4. Other Factors

**Figure 1:**
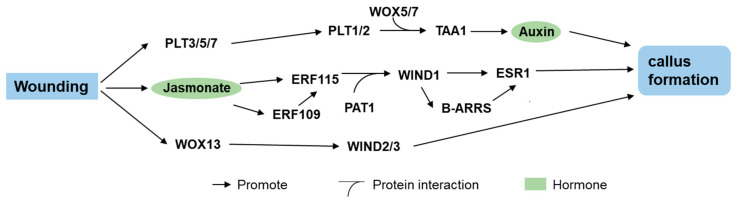
Molecular basis of callus formation regulated by wounding signaling in *Arabidopsis*. Wounding activates jasmonate (JA) signaling, which employs ETHYLENE RESPONSE FACTORS (ERF) transcription factors to upregulate *WOUND-INDUCED DEDIFFERENTIATION1* (*WIND1*). Concurrently, wounding induces the expression of *PLETHORAs* (*PTLs*) and *WUSCHEL-RELATED HOMEOBOX13* (*WOX13*). WOX13 subsequently promotes callus formation by directly activating *WIND2* and *WIND3*. Furthermore, *PLTs* enhance auxin biosynthesis through upregulation of *L-TRYPTOPHAN AMINOTRANSFERASE OF ARABIDOPSIS1* (*TAA1*).

Several genes have been identified as promising candidates for functioning in wound-induced callus formation. For example, *LATERAL ORGAN BOUNDARY DOMAIN16* (*LBD16*), *WOX5*, *WOX13*, *ETHYLENE RESPONSE FACTOR109* (*ERF109*), *ERF113*/*RAP2.6L*, *ERF114*, *ERF115*, and *OBP1* are rapidly induced after wounding, whereas *LBD29*, *WOX4*, *WOX14*, and *OBP4* are downregulated [88]. Wounding triggers jasmonate (JA) signaling, which acts through *ERF* transcription factors to regulate callus formation and shoot regeneration [92]. ERF115 and its interaction partner PHYTOCHROME A SIGNAL TRANSDUCTION1 (PAT1) play key roles in wound-induced callus formation and induce *WIND1* expression [88,93]. *WOX13* induced by *WIND1* directly upregulates the expression of *WIND2* and *WIND3* to promote callus growth [94]. To summarize this complex regulatory network, a graphical summary of the regulatory network of wound-induced callus formation in *Arabidopsis* is presented (Figure 1). Therefore, future research should prioritize the functional characterization of these genes to elucidate their roles in promoting callus formation and shoot regeneration.

## 4. Hormone Signaling Factors

**Figure 2:**
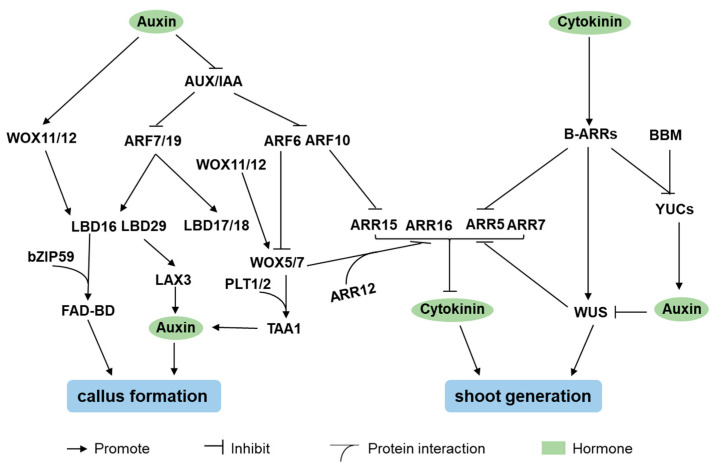
Molecular basis of callus formation and shoot regeneration regulated by hormone signaling in *Arabidopsis*. On callus-inducing medium (CIM), auxin triggers the degradation of AUXIN/INDOLE-3-ACETIC ACIDs (Aux/IAAs) to activate AUXIN RESPONSE FACTORs (ARFs). *ARFs* regulate callus formation through *LATERAL ORGAN BOUNDARY DOMAINs* (*LBDs*), *WUSCHEL-RELATED HOMEOBOX5* (*WOX5*), and *WOX7*. Additionally, auxin also induces the expression of *WOX11* and *WOX12*, which control callus formation via *LBD16*, *WOX5*, and *WOX7*. On shoot-inducing medium (SIM), cytokinin (CK) promotes shoot regeneration via B-type RESPONSE REGULATORs (ARRs), which directly and indirectly control *WUS* expression. B-type *ARRs* repress the expression of A-type *ARRs*, which are negative regulators in CK-regulated shoot regeneration.

Although auxin and CK are well established as central regulators of genetic transformation, recent evidence indicates that brassinosteroids (BRs), gibberellins (GAs), ABA, JA, and ethylene (ET) also participate in this process [10]. Nevertheless, the molecular mechanism by which auxin, CK, and JA regulate callus formation and shoot regeneration has been elucidated. The role of wound-induced JA was described in Wound-Inducible Factors (Section 3). This section will focus on how auxin and CK signaling regulate callus formation and shoot regeneration in *Arabidopsis* (Figure 2).

The AUXIN/INDOLE-3-ACETIC ACID (Aux/IAA) transcriptional repressors inhibit the activity of their interaction partners, AUXIN RESPONSE FACTOR7 (ARF7) and ARF19, which activate *LBD16*, *LBD17*, *LBD18*, and *LBD29* [95,96,97,98]. Concurrently, ARF10 and ARF16 suppress auxin accumulation by downregulating *WOX5* expression [99]. *LBDs*, important regulators of lateral root formation, induce callus formation in the absence of exogenous hormones and enhance regeneration capacity [97,98]. For instance, LBD16 interacts with *Arabidopsis* BASIC REGION/LEUCINE ZIPPER MOTIF 59 (AtbZIP59) to activate FAD-BINDING BERBERINE (FAD-BD) expression, regulating auxin-induced cell reprogramming during callus formation [100]. Similarly, LBD29 binds to the promoter of *LAX3*, an auxin-inducible auxin influx carrier, to regulate its expression [96]. Furthermore, *LBD16* and *LBD19* are upregulated by *WOX11* and *WOX12*, which are auxin-induced and directly activate the transcription of *WOX5* and *WOX7* [101,102]. *YUCCA* (*YUC*)-mediated auxin biosynthesis is essential for initiating cell fate transition [103,104]. The expression of *YUC3* and *YUC8* is regulated by *BBM* [105]. Finally, directional auxin transport mediated by PIN-FORMED (PIN) auxin transporters is critical for shoot regeneration, as demonstrated by the impaired regeneration in *pin* mutants [106,107].

In *Arabidopsis*, shoot regeneration is regulated by CK signaling through the coordinated action of B-type and A-type *RESPONSE REGULATORs* (*ARRs*), which function as positive and negative regulators, respectively [108,109,110,111,112,113]. B-type *ARRs* (including *ARR1*, *ARR2*, *ARR10*, and *ARR12*) promote shoot regeneration through multiple mechanisms. First, they directly activate the expression of *WUS* by binding to its promoter [108,114,115]. Second, B-type *ARRs* repress the expression of *YUC*, thereby reducing auxin accumulation and alleviating the auxin-mediated repression of *WUS* expression [114]. Third, B-type *ARRs* directly repress the expression of A-type *ARRs* to promote shoot regeneration [111]. In a parallel regulatory module, ARR12 interacts with WOX5 and WOX7 to repress A-type *ARRs* expression [39]. Finally, *WUS* directly suppresses A-type *ARR* expression, establishing a negative feedback loop in CK signaling [116].

## 5. Epigenetic Modification Factors

Genome-wide chromatin remodeling occurs throughout *in vitro* tissue culture, with epigenetic changes regulating cell fate transitions during dedifferentiation and regeneration [117,118]. Epigenetic modifications, such as DNA methylation, histone acetylation, and H3K4me3 and H3K27me3 deposition, are involved in genetic transformation.

Epigenetic modification factors play roles in callus formation and shoot regeneration by regulating auxin signaling and CK signaling. In auxin signaling, H3K9me3 demethylase JUMONJI C DOMAIN-CONTAINING PROTEIN30 (JMJ30) and H3K36me3 demethylase ARABIDOPSIS TRITHORAX-RELATED 2 (ATXR2) form a complex with ARF7 and ARF19 [119,120]. This ARF-JMJ30-ATXR2 complex activates *LBD16/29* expression by removing repressive H3K9me3 and H3K36me3 marks at their promoters [120]. In CK signaling, ATXR2 interacts with ARR1 to deposit H3K36me3 at the promoters of A-type *ARRs* (*ARR5* and *ARR7*), activating their expression. Since A-type ARRs are negative regulators, this ultimately leads to the repression of CK signaling and consequently downregulates *WUS* expression [119,120,121]. The auxin-inducible *ATXR2* gene is upregulated on CIM but downregulated on SIM. Functionally, the ARF-JMJ30-ATXR2 complex promotes callus formation on CIM then switches to an ATXR2-ARR1 module to promote shoot regeneration on SIM, indicating that the biological functions of *ATXR2* depend on hormonal signals in the culture medium [121,122]. Thus, beyond the specific roles of JMJ30 and ATXR2, other DNA methylation and histone modification factors directly and indirectly modulate *WUS* expression to regulate shoot regeneration [123,124].

MicroRNAs (miRNAs) repress gene expression primarily by targeting mature messenger RNAs (mRNAs) for degradation or translational repression. Multiple miRNAs have been shown to regulate callus formation and shoot regeneration by directly or indirectly targeting key genes of auxin and CK signaling. For instance, the miR156-targeted SQUAMOSA PROMOTER BINDING PROTEIN-LIKE (SPL) reduces shoot regenerative capacity by interacting with ARR2, a positive regulator of CK response [125]. Similarly, miR167 negatively regulates shoot regeneration via downregulating the expression of its target *ARF6* and *ARF8*, auxin response factors [126,127]. In contrast, miR160 inhibits callus formation by repressing its target *ARF10*, which functions as a repressor of *ARR15* expression via binding to its promoter [128]. Meanwhile, miR319-targeted TEOSINTE BRANCHED 1, CYCLOIDEA, and PROLIFERATING CELL NUCLEAR ANTIGEN BINDING FACTOR3/4 (TCP3/4) inhibit shoot regeneration by directly upregulating *ARR16* via binding to its promoter [129]. Furthermore, miR159, miR165, and miR166 collectively regulate callus initiation and shoot regeneration by fine-tuning phytohormone signaling through the repression of their respective target genes [30].

## 6. Conclusions and Future Perspectives

Genetic transformation is a powerful tool for studying gene function and crop improvement. The aforementioned summary demonstrates the feasibility of improving genetic transformation efficiency and overcoming genotype and explant dependency through the application of development factors (Table 1). Among all developmental factors, *GRF-GIF* chimera, *BBM-WUS* chimera, *WIND1*, *WOXs*, *DOFs*, and REF play important roles in improving transformation efficiency. Many of these factors, such as *GRF-GIF* chimera, *BBM-WUS* chimera, *AtGRF5*, and *AtWIND1*, have been successfully constructed into vectors and utilized in practical studies [59,130]. Notably, *TaWOX5* and *ZmBBM-WUS* not only improved transformation efficiency but also overcame genotype dependency [21]. *TaWOX5* enhanced the transformation efficiency of 29 wheat varieties, including previously non-transformable varieties (Bs366, Ningchun4, Aikang58, Xinong979, and Sunstate) and a landrace variety Luohanmai. *ZmBBM-WUS* also improved the transformation efficiency of the typically recalcitrant maize variety Zheng58 [53,85]. Furthermore, the combination of *GRF4-GIF1* and *ZmBBM-WUS2* overcame the barrier of mature embryo transformation, enabling the recovery of transgenic plants in the wheat varieties Kenong199 and Fielder [54].

With the application of developmental factors, a significant gap in transformation efficiency persists between most major crops and rice. In rice, the transformation efficiency using mature embryos achieved 97.0% in Japonica cultivars and 69.0–83.0% in Indica cultivars, independent of these development factors [131]. In contrast, transformation efficiency reached 46.2% for soybean using mature embryos, 37.5% for maize using immature embryos, and 90.0% and 19.4% for wheat using immature and mature embryos, respectively [21,85,87]. These findings reveal many challenges that require further investigation in the genetic transformation of major crops. For instance, soybean transformation is primarily hampered by low efficiency and genotype dependency, while in wheat, the transformation of mature embryos remains particularly inefficient. Most notably, the challenges in maize are even more complex, as efforts must concurrently address low efficiency, genotype dependency, and the development of efficient transformation protocols for mature embryos.

To grapple with these challenges, it is necessary to elucidate the underlying molecular mechanisms and identify key developmental regulators that enhance transformation efficiency. Currently, research on many developmental factors in enhancing transformation efficiency are largely confined to one species. This is particularly true for developmental factors involved in wound signaling, epigenetic modification, and hormone signaling mainly explored in *Arabidopsis*. Given that even evolutionarily conserved genes exhibit significant species-specific variation in their effects on transformation efficiency, it is essential to systematically characterize the function of their homologs in other plants. Beyond *Arabidopsis*, molecular mechanisms controlling genetic transformation remain underexplored in most species and require extensive investigation. Further research should also focus on characterizing the regulatory pathways governed by these efficient developmental factors. Recent advances in multi-omics technologies provide powerful tools to systematically decode the complex regulatory networks. For instance, by applying techniques such as RNA-seq, ATAC-seq, and CUT&Tag, a recent study revealed that expression patterns of some regeneration factors and the transcriptional regulatory routes of regeneration differ between wheat and *Arabidopsis* [73]. Furthermore, the study identified *TaDOF5.6* and *TaDOF3.4* as key developmental factors that significantly enhance transformation efficiency.

Genetic transformation in plants is fundamentally a cellular process [16,132,133]. At present, single-cell RNA sequencing (scRNA-seq) technology has enabled studying the mechanism of pluripotency acquisition in callus. This technology allows for direct comparison between regenerative-competent and non-competent cells, thereby facilitating precise dissection of the molecular mechanisms [39]. Meanwhile, as with other agronomic traits, transformation efficiency can be studied using both bi-parental populations and natural populations through approaches such as genome-wide association studies (GWAS), linkage analysis, and bulked segregant analysis (BSA) for candidate gene identification. Advances in artificial intelligence (AI) are further accelerating the identification of candidate regulators affecting regeneration capacity through the integration of machine learning with multi-omics data [3]. Furthermore, AI-driven prediction tools such as AlphaFold enable the rational design of optimized alleles that enhance the activity of key developmental factors [134]. This approach offers promise for overcoming genotype and explant limitations through engineering improved versions of existing developmental factors. Based on these insights, future research should employ integrated multi-omics sequencing and AI modeling to systematically elucidate species-specific regulatory networks governing plant regeneration. Such efforts are critical for developing effective strategies to enhance transformation efficiency and overcome the current bottlenecks in plant genetic transformation.

## Figures and Tables

**Table 1:** Functions of developmental factors in genetic transformation.

Developmental Regulators	Molecular Functions	Name	Species of Origin	Species of Application	Explants	Biological Functions	References
WUSCHEL-related homeobox 5 (WOX5)	maintaining stem cell identity in SAM and RAM	*TaWOX5*	wheat (*Triticum aestivum*)	wheat (*Triticum aestivum*)	immature embryo	2.1–21.4-fold increase in transformation efficiency	[21]
		*TaWOX5*	wheat (*Triticum aestivum*)	wheat (*Triticum aestivum*)	immature embryo	2.7-fold increase in transformation efficiency	[54]
		*ZmWOX2a*	maize (*Zea mays*)	maize (*Zea mays*)	immature embryo	improving transformation in recalcitrant variety B73	[40,41]
		*MdWOX11*	apple (*Malus domestica*)	apple (*Malus domestica*)	leaves	suppressing adventitious shoot formation	[42]
BABY BOOM-WUSCHEL (BBM-WUS)	BBM promotes cell proliferation and morphogenesis during embryogenesis; WUS maintains stem cell identity in SAM and RAM	*ZmBBM-WUS2*	maize (*Zea mays*)	wheat (*Triticum aestivum*)	immature embryo	2.8-fold increase in transformation efficiency	[54]
		*ZmBBM-WUS2*	maize (*Zea mays*)	maize (*Zea mays*)	immature embryo	2.5-fold increase in transformation efficiency	[53]
		*ZmBBM-WUS2*	maize (*Zea mays*)	maize (*Zea mays*)	mature embryo	improving transformation with mature embryo	[48]
		*ZmBBM-WUS2*	maize (*Zea mays*)	sorghum (*Sorghum bicolor*)	immature embryo	improving transformation efficiency	[48,52]
GROWTH-REGULATING FACTOR- GRF-INTERACTING FACTOR (GIF-GIF)	promoting cell proliferation during organogenesis	*AtGRF5*	*Arabidopsis*	cassava (*Manihot esculenta*)	leaf petiole	improving shoot regeneration	[64]
		*AtGRF5*	*Arabidopsis*	sugar beet (*Beta vulgaris* ssp. *vulgaris*)	leaves	improving shoot regeneration	[65]
		*BvGRF5-LIKE*	sugar beet (*Beta vulgaris* ssp. *vulgaris*)	sugar beet (*Beta vulgaris* ssp. *vulgaris*)	leaves	no	[65]
		*AtGRF5*	*Arabidopsis*	soybean (*Glycine max*)	mature embryo	improving shoot regeneration	[65]
		*AtGRF5*	*Arabidopsis*	sunflower (*Helianthus annuus*)	cotyledon	improving shoot regeneration	[65]
		*AtGRF5*	*Arabidopsis*	canola (*Brassica napus*)	hypocotyl	improving callus formation	[65]
		*AtGRF6*	*Arabidopsis*	canola (*Brassica napus*)	hypocotyl	improving callus formation	[65]
		*AtGRF9*	*Arabidopsis*	canola (*Brassica napus*)	hypocotyl	improving callus formation	[65]
		*BnGRF5-LIKE*	canola (*Brassica napus*)	canola (*Brassica napus*)	hypocotyl	improving callus formation	[65]
		*ZmGRF5*	maize (*Zea mays*)	maize (*Zea mays*)	immature embryo	improving shoot regeneration	[65]
		*AtGRF5*	*Arabidopsis*	maize (*Zea mays*)	immature embryo	improving shoot regeneration	[65]
		*TaGRF4-GIF1*	wheat (*Triticum aestivum*)	wheat (*Triticum aestivum*)	immature embryo	3.7-fold increase in transformation efficiency	[54]
		*GRF-GIF*	grape (*Vitis vinifera*)	*citrus*	epicotyl	4.7-fold increase in shoot regeneration	[61]
		*GRF-GIF*	*citrus*	*citrus*	epicotyl	4.7-fold increase in shoot regeneration	[61]
		*GRF4/8-GIF1*	tomato (*Solanum lycopersicum*)	lettuce *(Lactuca* spp.)	cotyledon	improving shoot regeneration	[66]
		*GRF4-GIF1*	pepper (*Capsicum annuum*)	lettuce (*Lactuca* spp.)	cotyledon	improving shoot regeneration	[66]
		*GRF4-GIF1*	*citrus*	lettuce (*Lactuca* spp.)	cotyledon	improving shoot regeneration	[66]
		*GRF4-GIF1*	grape (*Vitis vinifera*)	lettuce (*Lactuca* spp.)	cotyledon	improving shoot regeneration	[66]
		*GRF4-GIF1*	grape (*Vitis vinifera*)	cassava (*Manihot esculenta*)	leaf-petiole	improving shoot regeneration	[64]
		*GRF4-GIF1*	wheat (*Triticum aestivum*)	rice (*Oryza sativa*)	mature embryo	2.1-fold increase in transformation efficiency	[61]
		*GmGRF3-GIF1*	soybean (*Glycine max*)	soybean (*Glycine max*)	mature embryo	2.7-fold increase in transformation efficiency	[23]
		*ClGRF4-GIF1*	watermelon (*Citrullus lanatus*)	watermelon (*Citrullus lanatus*)	cotyledon	9.0-fold increase in transformation efficiency	[63]
		*TaGRF4-GIF1*	wheat (*Triticum aestivum*)	*Dendrobium catenatum*	young seedling	improving shoot regeneration	[62]
		*DcGRF4-GIF1*	*Dendrobium catenatum*	*Dendrobium catenatum*	young seedling	improving shoot regeneration	[62]
		*TaGRF4-GIF1*	wheat (*Triticum aestivum*)	wheat (*Triticum aestivum*)	immature embryo	7.8-fold increase in transformation efficiency	[61]
		*GRF4-GIF1* + *ZmBBM-WUS2*		wheat (*Triticum aestivum*)	immature embryo	5.2-fold increase in transformation efficiency	[54]
		*GRF4-GIF1* + *ZmBBM-WUS2*		wheat (*Triticum aestivum*)	mature embryo	transformation efficiency from 0% to 19.4%	[54]
		*GRF-GIF-BBM*	maize (*Zea mays*)	maize (*Zea mays*)	immature embryo	7.0-fold increase in transformation efficiency	[82]
DNA binding with one finger (DOF)	promoting cell proliferation	*TaDOF5.6*	wheat (*Triticum aestivum*)	wheat (*Triticum aestivum*)	immature embryo	1.9-fold increase in transformation efficiency	[73]
		*TaDOF3.4*	wheat (*Triticum aestivum*)	wheat (*Triticum aestivum*)	immature embryo	2.1-fold increase in transformation efficiency	[73]
WOUND-INDUCED DEDIFFERENTIATION1 (WIND1)	promoting cell dedifferentiation and proliferation	*ZmWIND1*	maize (*Zea mays*)	maize (*Zea mays*)	immature embryo	3.2-4.0-fold increase in transformation efficiency	[85]
		*AtWIND1*	*Arabidopsis*	canola (*Brassica napus*)	hypocotyl	improving shoot regeneration	[84]
		*AtWIND1*	*Arabidopsis*	*Arabidopsis*	young seedlings	improving shoot regeneration	[84]
REGENERATION FACTOR1 (REF1)	activating *WIND1* expression	*SIREF1*	tomato *(Solanum lycopersicum*)	tomato *(Solanum lycopersicum*)	hypocotyl	12-fold increase in transformation efficiency	[87]
		*GmREF1*	soybean (*Glycine max*)	soybean (*Glycine max*)	mature embryo	5-fold increase in transformation efficiency	[87]
		*ZmREF1*	maize (*Zea mays*)	maize (*Zea mays*)	immature embryo	4-fold increase in transformation efficiency	[87]
		*TaREF1*	wheat (*Triticum aestivum*)	wheat (*Triticum aestivum*)	immature embryo	4-fold increase in transformation efficiency	[87]

SAM, shoot apical meristem; RAM, root apical meristem.

## Data Availability

Data are contained within the article.

## Abbreviations

The following abbreviations are used in this manuscript:

GA	Gibberellin
CK	Cytokinin
ABA	Abscisic Acid
BR	Brassinosteroid
JA	Jasmonate
ET	Ethylene
